# The House of Steinway

**Published:** 1875-03

**Authors:** 


### THE HOUSE OF STEINWAY.

The individual who examines, however critically, one of the grand instruments produced at the establishment of the Steinways, will be surprised to learn that there are forty-two distinct lines of business therein represented. Very few houses embody all, or any large proportion, of these various trades within themselves. The small makers buy the various parts in limited quantities, put them together, and call themselves "pianoforte manufacturers." Any man who has money enough to buy the parts of one instrument, and who knows enough to put these parts into the usual form, however imperfectly, may boldly fling his standard to the breeze.

But he labors under great disadvantages. He has bought whatever the dealers in "parts" happened to have for sale, and he has put those incongruous materials together as best he could. He can not reject a piece of wood or a casting because it is not quite perfect, because he can not afford to do it; he can not secure the most costly and skillful labor, because he lacks the immense capital required. His instrument is conceived in imperfection, and is given to the world in an imperfect state. It can never confer the highest gratification upon those who listen to its notes. It can never be looked upon as a valid and substantial "piece of property," for it lacks the enduring qualities demanded.

If you visit the factory of the Steinways, you will notice that one of the last touches given to a piano is the addition of its soul—the sounding-board. Without this thin, clear sheet of spruce, under the strings, the piano would be a dead and useless box of wood and wire. To secure the best material for this resonant film, almost every substance in nature has been tried, but nothing has been found to equal spruce. Countless experiments have been made with a view to ascertain precisely the best way of shaping, arranging, and fixing the sounding-board,—the best thickness, the best number and direction of the supporting ribs; and here the genius of the artist is most completely taxed. Here it is that the Steinway piano excels others; here it is that the marvellous tones which make that instrument peerless, are secured.

After the sounding-board is delicately adjusted the strings are inserted; then the action and the keys. Every one will pause to admire the hammers of the piano, so light yet so capable of giving a telling blow,—which evolve all the music of the strings, but mingle with that music no click, nor thud, nor thump, of their own. The felt with which these hammers are faced, is, for some of the hammers of a Steinway piano, over an inch thick, and costs more than five dollars a pound in gold. Only Paris, it seems, can make it good enough for the purpose. Some of the keys have a double felting, compressed from an inch and a half to three quarters of an inch; and others again have an outer covering of leather, to keep the strings from cutting the felt. Simple as the finished hammer looks, there are a hundred and fifty years of thought and experience in it. It required half a century to exhaust the different kinds of wood, bone, and cork; and when, about 1760, the idea was conceived of covering the hammers with something soft, another century was to elapse before all the leathers and fabrics had been tried, and woollen felt found to be superior to all else.

The piano completed, the last polish given it by the bare hand of the workman, it is taken to a very important personage—the "regulator." He inspects, rectifies, tunes, harmonizes, and perfects the whole. He refines, elevates, exalts the beautiful creation; and when he looks upon the work of so many hands, and pronounces it good, there is nothing more to be said. Summon now your experts, your artists, your great musicians, and let the listening world hear these dulcet notes! Lo, a new Steinway piano, filled with enchanting music, overflowing with its sentient, harmonious life, awaits the buyer!

